# Cooled radiofrequency ablation versus cryoneurolysis of the genicular nerves for the symptomatic pain management in knee osteoarthritis: a study protocol of a prospective, randomized, single-blinded clinical trial

**DOI:** 10.1186/s13018-023-03737-1

**Published:** 2023-04-12

**Authors:** A. Panagopoulos, P. Tsiplakos, K. Katsanos, P. Antzoulas, J. Lakoumentas

**Affiliations:** 1 Orthopaedic Department, Patras University Hospital, University of Patras, Patras, Greece; 2 Department of Interventional Radiology, Patras University Hospital, University of Patras, Patras, Greece; 3 Department of Medical Physics, School of Medicine, Patras University, Patras, Greece

**Keywords:** Knee osteoarthritis, Genicular nerves, Pain, Cryoneurolysis, Cooled radiofrequency ablation

## Abstract

**Background:**

Cooled radiofrequency ablation (CRFA) and cryoneurolysis (CRYO) are two novel methods of genicular neurolysis to relief pain in symptomatic knee osteoarthritis (KOA). In this study, the two methods will be compared, giving us the opportunity to investigate their efficacy, safety and complications.

**Methods:**

In this prospective randomized trial 70 patients with KOA will be recruited using a diagnostic block of four genicular nerves. Two groups will be created through software randomization: a CRFA group (35 patients) and a CRYO group (35 patients). The target of the interventions will be four genicular nerves; the superior medial, superior lateral, inferior medial, as well as the medial (retinacular) genicular branch from vastus intermedius. The primary outcome of this clinical trial will be the efficacy of CRFA or CRYO at 2-, 4-, 12-and 24-weeks post-intervention using the Numerical Rating Pain Scale (NRPS). The secondary outcomes are the safety of the two techniques, as well as the clinical evaluation using the Knee Injury and Osteoarthritis Outcome Score (KOOS), the Oxford Knee Score (OKS), and the 7-point scale of Patient Global Impression of Change (PGIC).

**Discussion:**

These two novel techniques can block pain transmission through genicular nerves in different ways. In contrast to cryoneurolysis, the CRFA method has been well documented in the past. This is the first clinical trial to compare CRFA vs CRYO and draw conclusions about their safety and efficacy.

***Trial registration*:**

ISRCTN87455770 [https://doi.org/10.1186/ISRCTN87455770]. Registered 29/3/2022, first patient recruited 31/8/2022.

## Introduction

Knee osteoarthritis (KOA) is a degenerative joint disease that is mainly characterized by damage and loss of articular cartilage, remodeling of the subchondral bone, osteophyte formation, ligamentous laxity, weakening of periarticular muscles, and, in some cases, synovial inflammation [1]. According to Global Burden of Disease (GBD-2015 study), approximately 85% of the burden of osteoarthritis worldwide is associated with KOA [2], which has shown increased prevalence, multimorbidity, and a higher number of drug prescriptions [3]. Total knee arthroplasty (ΤΚΑ) is an effective treatment option for end-stage knee arthritis and persistent severe pain [4], but still 15–20% of the patients remain dissatisfied following this procedure; overestimated pre-operative expectations, a positive history of mental health problems, history of low back pain, and severe post-operative pain and/or suboptimal post-operative physical function are considered important factors for patient dissatisfaction following TKA [5]. However, the relatively slow progression of the knee osteoarthritis allows for stepwise algorithmic approach using non-surgical or non-pharmacological treatment options [6, 7]. Through the wide array of non-surgical treatment options, cooled radiofrequency ablation (CRFA) and cryoneurolysis (CRYO) have been proposed.

Radiofrequency ablation (RFA) is a process of thermal nerve degradation using a probe that provides radiofrequency energy. CRFA uses a water-supply system to cool the RFA probe internally. While internally cooled probes operate at a temperature of 60 °C, the temperature of the surrounding tissues reaches 80 °C, thus providing a larger lesion around the probe [8]. CRFA delivers larger and more spherical lesions compared to conventional radiofrequency ablation, thus increasing the likelihood of ablating targeted nerves; subsequently, CRFA delivers a significantly higher energy to surrounding neural tissues, which has been hypothesized to result in more durable clinical outcomes [9]. This procedure aims to disrupt the transmission of pain signals from the osteoarthritic knee via the genicular nerves. Traditionally, CRFA studies are primarily targeting three genicular nerves: the superior lateral (SLGN), superior medial (SMGN), and inferior medial (IMGN). Some reports also include the medial (retinacular) genicular nerve from vastus intermedius (MRGN) [10, 11].

Cryoneurolysis involves the application of cold temperatures (− 20 to − 100 °C) to a peripheral nerve, leading to Wallerian degeneration and subsequent analgesia, while the nerve retains its ability to regenerate [12–16]. In contrast to CRFA which completely ablate the targeted nerve, CRYO has the ability to disrupt nerve function while structural elements of the nerve bundle remain intact. This allows for complete regeneration and functional recovery of the nerve over time. CRYO studies, so far, are primarily targeting the infrapatellar branch of the saphenous nerve (IPBSN), a sensory nerve that innervates the anterior and inferior parts of the knee capsule and/or the anterior femoral cutaneous nerve (AFCN) [13, 14, 16]. The CRYO probe that will be used in this study (IceSphere 1.5 CX, Galil Medical Ltd) creates also a spherical lesion of ice around the tip thus increasing the likelihood of degenerate the targeted nerves.

Clinical evidence in general, suggests that both CRFA and CRYO are safe and effective procedures for the symptomatic management of KOA pain [11–18].

The primary objective of our proposed trial is to evaluate the efficacy of CRFA or CRYO at 2-, 4-, 12- and 24-weeks post-intervention in patients with painful KOA using the Numerical Rating Pain Scale (NRPS).

The secondary objectives are the comparison of safety and tolerability of the two interventions, as well as the patient’s clinical outcome at 12- and 24-weeks post-intervention, using the Knee Injury and Osteoarthritis Outcome Score (KOOS) and the Oxford Knee Score (OKS). Also, the 7-point scale of Patient Global Impression of Change (PGIC) will be used at 2-, 4-, 12-, and 24-weeks post-intervention to capture the patients’ feelings of improvement.

## Methods

**Fig. 1:**
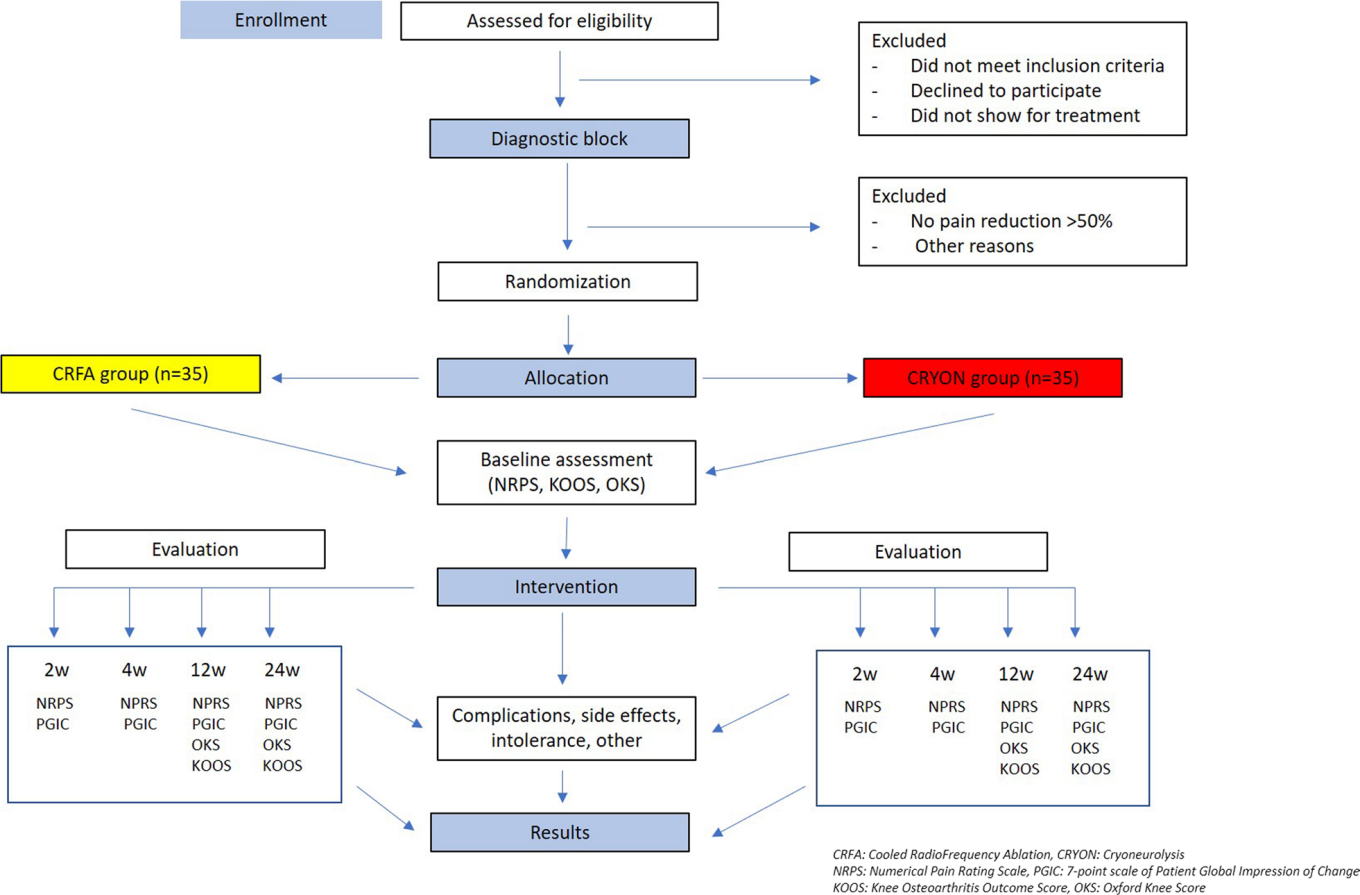
Flowchart of the study

This study protocol describes the design of a prospective, single-blinded RCT with an allocation ratio of 1:1, between CRFA and CRYO. Our main hypothesis is that substantial relief of pain would be achieved with both techniques (CRFA and CRYO) compared to baseline values and that both methods would be effective and tolerable in the intermediate management of KOA. (Fig. 1).

### Study setting

The study will be conducted at the Department of Orthopaedics, University Hospital of Patras, Greece. The institutional review board (IRB) of our University Hospital has approved the study (11846/05/10/2021), and written informed consent from participating patients will be obtained. The study will be conducted in accordance with all applicable laws and regulations as specified in the International Conference on Harmonization Guideline for Good Clinical Practice and the Declaration of Helsinki.

### Eligibility criteria

After establishing the diagnosis of knee osteoarthritis according to the eligibility criteria outlined below, the patients will visit the Department for initial clinical assessment and diagnostic block of the genicular nerves**.** Patients will be asked to rate the percentage of their knee pain according to NPRS while performing ambulation, squatting, and other maneuvers that typically provoke their pain during the next 30 min in the office. After the diagnostic block of the six main anatomical sites described below the patients will then be asked to rate any reduction in their knee pain while performing the same maneuvers in the office. The primary investigators will rate these results and decide on their eligibility. Patients will be only enrolled in the study if they report, at least 50% reduction in pain (as measured in the NRPS). Eligible patients will be then randomizing as 1:1 to either CRFA or CRYO. Participants with bilateral KOA will not be excluded; only one knee will be screened and enrolled as the “index knee” for treatment.

Patients of either sex can participate in the clinical trial if they have:

1. the NICE clinical criteria [19] of primary KOA for one or both knees: as follows: (a) age > 45; (b) activity-related joint pain and (c) no morning joint stiffness or morning stiffness that lasts no longer than 30 min.
2. Radiological confirmation of knee arthritis (grade ≥ 2) according to the Kellgren and Lawrence classification [20].
3. Chronic knee pain for a minimum duration of 6 months.
4. Pain intensity ≥ 4 on the (NRPS).
5. A decrease of ≥ 50% in NRPS scores with diagnostic genicular nerve block.
6. The ability to communicate in Greek.

### Exclusion criteria

Patients who belong to any of the following groups will be excluded:

1. Inflammatory or posttraumatic knee arthritis
2. Patients who received CRYON or CRFA treatment in the past
3. Injection of hyaluronic acid, PRPs, or corticosteroids within the previous 3 months
4. Significant structural deformities affecting locomotion and knee function aside
5. from osteoarthritis and which might cause chronic knee pain
6. Body mass index ≥ 40 kg/m^2^
7. Uncontrolled serious diseases (cancer, diabetes, end-stage heart disease, etc.)
8. Unstable psychiatric illnesses
9. Coagulopathy or bleeding disorders
10. Active systemic or local infections
11. Disease associated with reactions to the cold, such as cryoglobulinemia

Patients who fulfill the inclusion criteria will be then informed about the proposed treatment options (CRFA or CRYO). The current literature on CRFA has shown promising results for pain relief up to 12 months (11,17,18), whereas data are scarce for CRYO (13,14,16) which has been less investigated thus far. Treatment with cryoneurolysis is still in its early stage and further studies are needed to determine methodological strategies optimizing its potential therapeutic effects. Nyggard et al. [14] have recently (2021) published a study protocol that aims to compare cryoneurolysis + standardized education and exercise vs sham group + standardized education and exercise; in this study the ICEfx technology will be used, as in our study, but the targeting nerves would be different (the infrapatellar branch of the saphenous nerve (IBSN) and the anterior femoral cutaneous nerve (AFCN)). The extent of nerve damage depends on several factors including temperature, contact area, freezing rate, exposure time and thawing strategy. Our study would apply a specific freezing protocol, similar to Nyggard et al. [14], that is relatively short (3 min), utilizing a single freezing cycle and not at full effect (slower freezing). This might reduce potential risks associated with the procedure but might also attenuate treatment effects.

### Intervention description

#### Initial assessment

The baseline assessment will include: (a) the medical history of the patient (age, sex, body mass index, duration of pain, comorbidities, use of analgesics, and prior interventions of the knee joint); (b) radiological classification using standing AP and lateral x-rays of the knee joint and (c) baseline clinical evaluation using the NRPS, OKS, and KOOS. The OKS is a 12-item patient-reported outcome score, specifically designed and developed to assess function and pain after total knee replacement (TKR) surgery [21]. It is valid, reproducible, and sensitive to clinically important changes without a ceiling or floor effect for both its pain and function subscales [22]. The OKS has been translated and validated for Greek patients with knee osteoarthritis [23]. The KOOS questionnaire was developed in the 1990s as an instrument to assess the patient’s opinion about their knee and associated problems [24]. KOOS score has 5 subscales; Pain, other Symptoms, Function in daily living (ADL), Function in sport and recreation (Sport/Rec) and knee related Quality of life (QOL). KOOS includes WOMAC Osteoarthritis Index LK 3.0 in its complete and original format (with permission), and its convergent and divergent construct validity has been determined in multiple studies in comparison to several instruments including the different subscales of SF-36 and the Lysholm knee scoring scale [25]. The KOOS questionnaire has been translated and validated for Greek patients with total knee replacement [26].

Patients who fulfill the inclusion criteria and have no exclusion criteria will be informed about the clinical trial in detail, and a consent form will be required to proceed.

### Targeting of the nerves (for diagnostic block or final intervention)

Precise targeting of genicular nerves using fluoroscopic guidance is mandatory for a successful procedure. Most of the current CRFA studies are using the classic radiological targets proposed by Choi et al. [27], McCormick et al. [28] and Conger et al. [29] aiming for the SLGN and SMGN at the junction of the midpoint of the femoral shaft and the lateral/medial femoral condyle whereas for the IMGN at the junction of the midpoint of the tibial shaft and medial tibial condyle. However, recent anatomic and dissection studies have suggested different target points especially for the SLGN and SMGN [30–33] and have proposed supplementary or totally different landmarks for precising targeting.

**Fig. 2:**
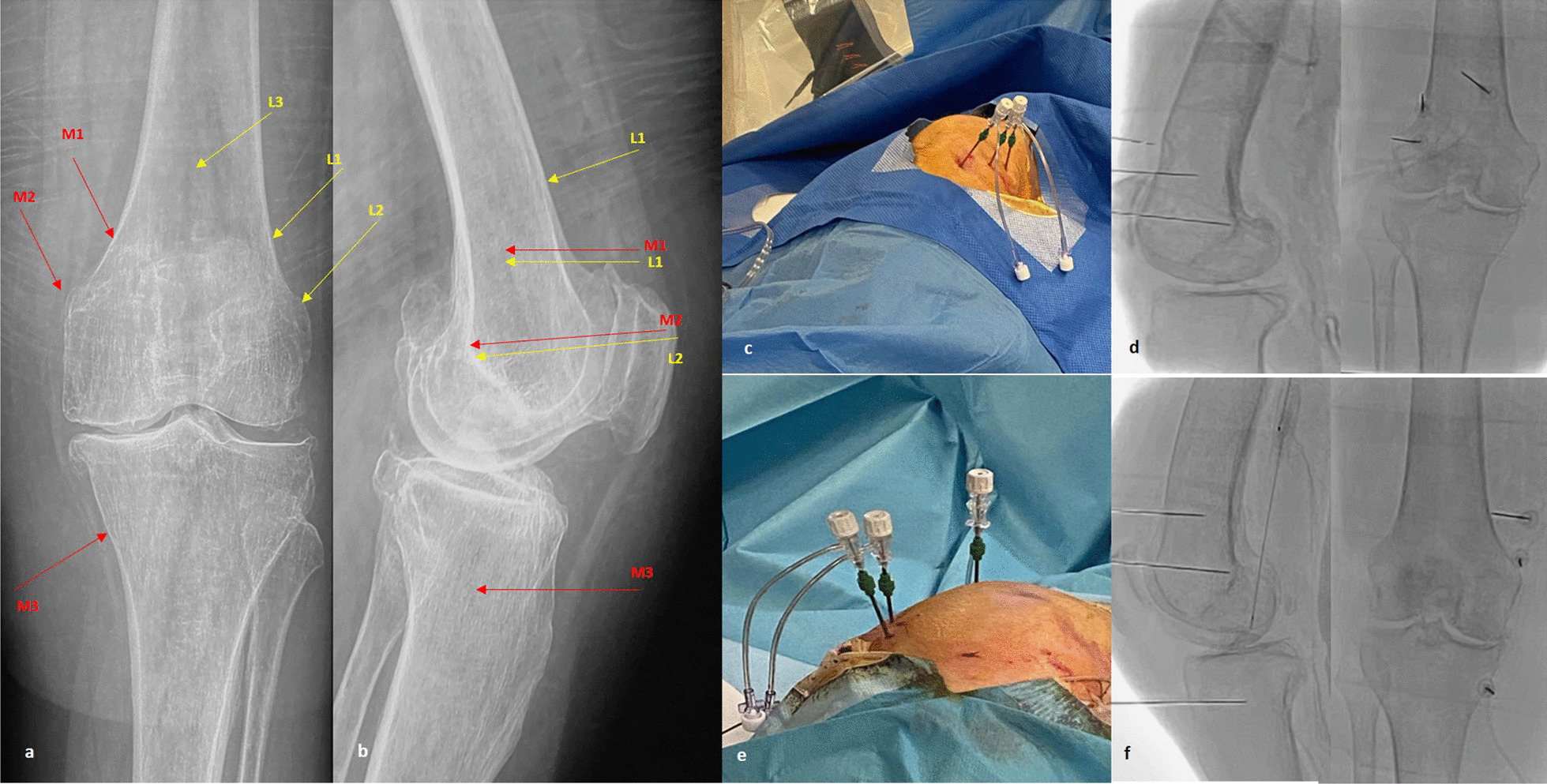
**a, b** Anteroposterior and lateral radiographs of an arthritic knee showing the intended target-points of the genicular nerves (M1, M2 = SMGN, M3 = IMGN, L1, L2 = SLGN, L3 = MRGN); **c** intraoperative picture of the needles in the lateral side just prior to ablation **d** anteroposterior and lateral fluoroscopic images of the exact location of the needles in the lateral side; **e** intraoperative picture of the needles in the medial side just prior to ablation; **f** anteroposterior and lateral fluoroscopic images of the exact location of the needles in the medial side

In the current study the target of the diagnostic block or final intervention will be the SMGN, the SLGN, and the IMGN, as well as the medial (retinacular) genicular branch from vastus intermedius (MRGN) (Fig. 2). For the SLGN we will use two landmarks: the classical one at the confluence of the femoral shaft and the lateral femoral condyle in the AP view and the midpoint of the femur width in the lateral view [27–29] but also the new proposed landmark from Fonkoue et al. [31] that is located more distal (closer to lateral epicondyle) in the AP view and laterally in the area connecting the posterior cortex of the femur shaft and the superior edge of the lateral condyle. Similarly, for the SMGN the classic point at the confluence of the femoral shaft and the medial femoral condyle in the AP view and the midpoint of the femur width in the lateral view will be used first followed by the new landmark [31] located more distal and closer to adductor tubercle (AT) in the AP view and just few millimeters anterior to the AT in the lateral view. The IMGN will be targeted at the concave transition between the tibial plateau and adjacent metadiaphyseal shaft in the AP view and at the midpoint of the tibia shaft in the lateral view [27–31]. Finally, the MRGN will be located at the middle of the AP distance of the femur, 3–4 cm above the superior patella pole, as has been described by Wong et al. [11].

For the nerve blocking procedure 1–2 ml of 2% lidocaine would be injected in the six different areas that mentioned previously, and the patients will then be asked to rate the percentage reduction in their knee pain while performing ambulation, squatting, and any other maneuvers that typically provoke their pain during the next 30 min in the office. Patients with a reduction in pain (> 50%) will proceed to the next phase, whereas those who experience no change in perceived pain will be excluded from the clinical trial.

### Intervention

Patients will be placed in the radiolucent table at the Department of Interventional Radiology in a supine position with a bolster under the knee to produce 30° of flexion; this position flattens the suprapatellar joint space, thus minimizing needle trespass, and also allows for an unobstructed lateral view of the knee. The treated knee will be draped and sterilized in a standard manner. Patients will be continuously monitored and administered conscious sedation (1–2 mg IV midazolam and/or fentanyl 25–100 mcg IV) and supplemental oxygen. Fluoroscopic images will be obtained to align the femur in an anteroposterior (AP) view in order to target the genicular nerves at locations above the femoral condyles and for the IMGN a greater caudal tilt of the fluoroscope will be obtained to square off the tibial plateau for an appropriate AP view. A sealed and opaque envelope will be brought to the operating theatre, dictating the group of each patient. The location of the nerves (SLGN, SMGN, IMGN, and MRGN) will be identified using fluoroscopy**,** according to previously described methods.

### CRFA technique

Two to 3 ml of 1% lidocaine is used to anesthetize the skin and subcutaneous tissues before cannula insertion at each target site under fluoroscopic guidance. Thereafter, a 50–150 mm 17-gauge introducer needle will be placed to ablate the SLGN, SMGN, IMGN, and MRGN. One milliliter of 2% lidocaine will be injected through the introducer needles to anesthetize the area before ablation. After placement of the introducer needle, a 18 gauge, internally cooled 4-mm active tip RFA electrode (Coolief, Halyard Health, Alpharetta, GA, USA) will be placed into the introducer needle, and the positioning is checked again using fluoroscopy. The introducer is connected to COOLIEF* Cooled RF Advanced Generator that allows staggered start, stop and adjusting of 4 independent channels. Motor nerve activity will be excluded by testing at 2 Hz and 1 mA. The CRFA probes will be advanced, and ablation will be performed with lesion settings at 60 °C (80–90 °C adjacent tissue temperature) for 2.5 min. As mentioned before, this type of ablation creates a spherical lesion at the tip of the probe. Cooled RF electrodes include a thermocouple at the active electrode tip to provide a temperature-controlled lesion formation. This involves water-cooling of the active electrode tip during the duration of the process without tissue charring at the electrode tip; this mechanism doubles the lesion radius and increase the lesion volume by 5 times.

#### CRYO technique

Two to 3 ml of 1% lidocaine is used to anesthetize the skin and subcutaneous tissues before probe insertion at each target site under fluoroscopic guidance. A cryoneurolysis probe (ICESphere 1.5 CX, Galil Medical Ltd.) will be inserted in the proximity of the six target points, guided by fluoroscopic visualization, as previously described. VisualICE generator (Galil Medical Ltd.) will be used for cryoneurolysis which utilizes Argon as a coolant and Helium to thaw. The procedure will be performed with a single freeze cycle: 30 s at an effect of 20% and 2 min 30 s at 60% effect. After freezing cycle, 1 min of active thaw and 1 min of passive thaw will be used. As mentioned before the CRYO probe creates a spherical lesion (ice-ball) that surrounds the active tip. The temperature gradient away from the probe increases importantly; the typical isotherm with the needle has a temperature of − 40 °C in an area equivalent to 15 × 23 mm. Already at 23 × 29 mm the temperature has risen to − 20 °C and at 33 × 37 mm the temperature is up to 0 °C. The thawing phase (2 min) is not considered active treatment but is necessary for the ice-ball to melt gradually for later safe removal of the probe. The active ablation time of CRYO (3 min) is similar to that of CRFA (2.5 min) but with a different mechanism. The area of ablation is spherical in both techniques thus increasing the probability that a targeted sensory nerve will be captured in the “sphere” of tissues neuroablated. Finally, the “slow” effect of freezing (20% for 30 s and 2 min 30 s at 60%) has been already proposed for genicular nerve ablation [14] in patients with KOA and is considered less destructive as it aims on Wallerian nerve degeneration than nerve tissue destruction. In other more severe cases (primary tumors, metastases) the CRYO technology is used at higher effect levels, larger areas of ablation with several probes inserted and also repeated cycles of freezing.

Patients will discontinue all pain medications (opioids, NSAIDS, anti-depressants and anti-convulsant drugs), supplements, chondroprotective drugs, and other alternative therapies for KOA for 10 days prior to the screening/baseline visit. During follow-up, patients will be prohibited from undergoing any other adjunctive treatment for KOA, including steroid injections, viscosupplementation, and pain medications. Serious local complications from either CRFA or CRYO and the need for medications for pain relief will be a criterion for study discontinuity.

### Outcomes

Patients will be assessed at baseline and 2-, 4-, 12-, and 24-weeks post-intervention (Table 1).

**Table 1:** Timeline of the study

Timepoint	Enrollment		Allocation		Assessment times		
	− 3 w	− 1 w	0 w	2w	4 w	12 w	24 w
Enrollment							
Eligibility screening	√						
Informed consent	√						
Genicular nerve block		√					
Allocation			√				
Outcome measures							
NPRS (primary)			√	v	√	√	√
KOOS			√	–	v	√	√
OKS			√	–	v	√	√
PGIC			–	–	v	√	√
Complications medications			Intra-op				
			√	v	√	√	√
			√	v	√	√	√

*NPRS* Numerical Pain Rating Scale, *KOOS* Knee Injury and Osteoarthritis Outcome Score, *OKS* Oxford Knee Scores, *PGIC* Knee Patient Global Impression of Change

The primary outcome would be the efficacy of CRFA or CRYO at 2-, 4-, 12- and 24-weeks using the Numerical Rating Pain Scale (NRPS). The secondary outcomes will be the potential improvement of the KOOS, and OKS at 12- and 24-weeks post-intervention. Patients will be also asked about their impression of improvement using the 7-point scale of Patient Global Impression of Change (PGIC) [34]; PGIC responders are the patients who indicate that they are either “very much improved” or “much improved” at each follow-up assessment. Expected side effects and complications (for example, bruising, swelling, numbness, inflammation, and/or erythema) involving percutaneous access to the nerves and the use of local anesthesia will be assessed at each follow-up visit and documented independently, except for loss of motor function outside the treatment area. The details of the timeline, assessments, and follow-up evaluations are presented in Table 1.

### Sample size

**Fig. 3:**
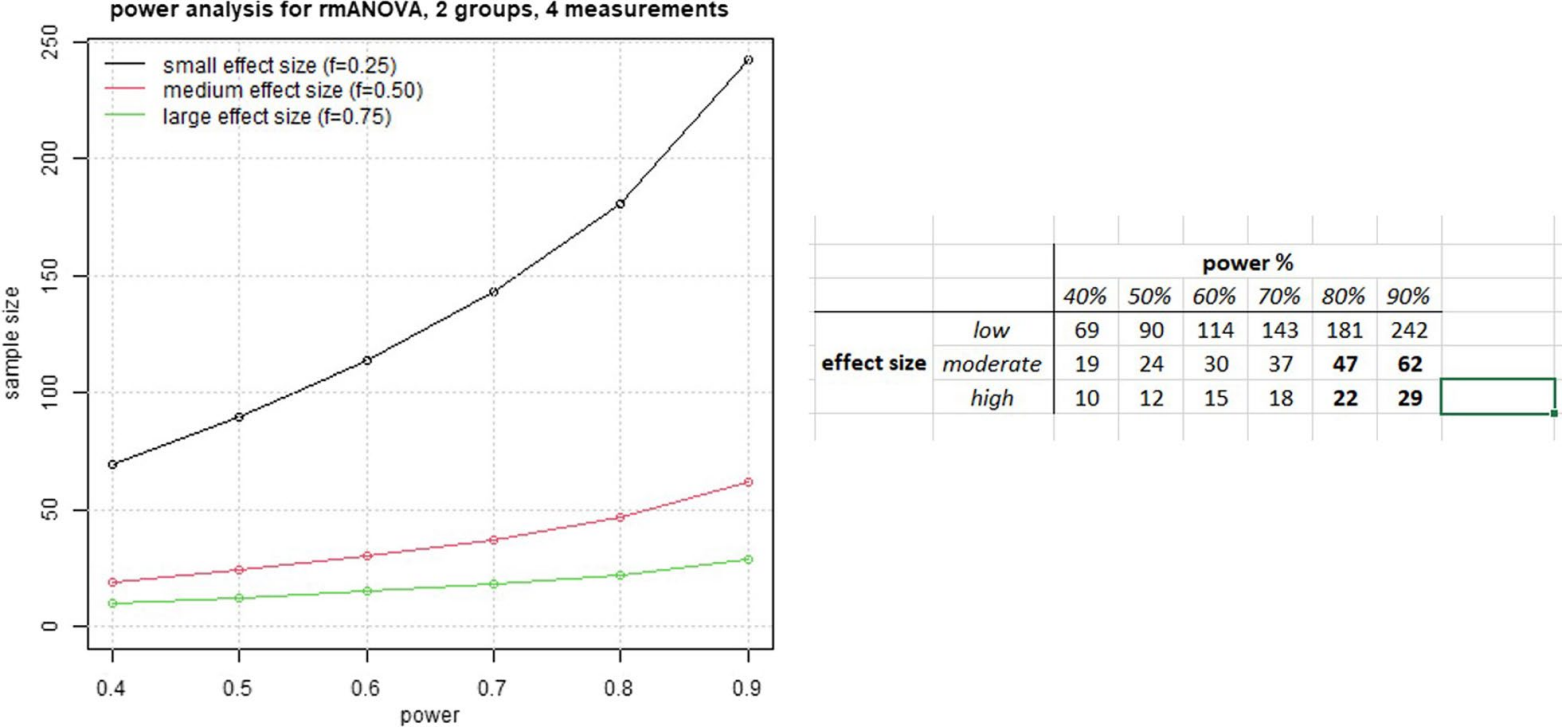
Diagram of the proposed power analysis for the 2 groups in 4 different assessment points using rmANOVA

According to the protocol, the patients will be randomly assigned into two groups, and they will all be evaluated (with NPRS) four more times after the baseline assessment (15 days, 4 weeks, 12 weeks, and 24 weeks, post-intervention). The main objective is to examine the possible diversity in NPRS (primary outcome) improvement among patients in different groups. The regular statistical approach to do this is to perform repeated measures ANOVA (rmANOVA), taking as given that all ANOVA assumptions are met (for example, normality of scores). Thus, the statistical power analysis for an rmANOVA procedure is as follows: we, as a priori, assume a number of groups equal to two and a number of measurements (except baseline) equal to four. More specifically, the NPRS is expected to be reduced in the two groups (CRYO and CRFA) at least 30%, relative to baseline assessment. According to Cohen [35], this fact denotes a medium effect size of approximately 0.50. Statistical significance is usually considered at the 5% level, and the power analysis curve for the rmANOVA is shown in Fig. 3. This figure illustrates that approximately 47 patients suffice for an achieved power of 80% using rmANOVA, taking into account a moderate effect size and 62 patients for 90% statistical power. Considering a 10% loss of follow up, we will enroll 70 patients in the study.

### Recruitment

Approximately 400 patients with symptomatic KOA visit our clinic annually. A small percentage of patients have end-stage disease and are only amenable to total knee replacement. Most remaining patients initially require conservative measures to control their symptoms, and among those, a respectable percentage has not been able to respond to oral medications, physiotherapy, loss of weight, and injectable therapies. This will be the pool of patients that will participate in our randomized trial. We estimate that we will be able to include 70 patients in the study in 10–12 months.

### Randomization—sequence generation

A total of 70 patients will be included in the study (CRFA:35; CRYO:35) Randomization sequence will be created using Stata 9.0 (StataCorp, College Station, TX) statistical software and will be stratified with a 1:1 allocation ratio using random block sizes of 2, 4, and 6. An investigator not involved in the main study (J.L.) will perform the randomization process. These data will be stored on computer and will be available at the day of the intervention.

### Concealment mechanism

For each surgical session including four patients, J.L. will seal patient details and the randomized treatment in an opaque envelope that will be given to the coordinated nurse just before the start of the intervention. The patients, relatives, investigators, nurses, and all relevant personnel would be blinded to the indented treatment. The envelope will be opened by the coordinated nurse after the patient is sedated and placed to the radiological table, the knee is properly prepared and the surgeons are sterile and ready to apply the intervention. Both generators for CRFA or CRYO would be available at the interventional radiological department. After opening the envelope, the procedure will be unblinded for all participants.

### Implementation and blinding

Enrolment of participants will be performed by the main investigators (A.P., P.T.) at the outpatient office of our department according to the eligibility criteria listed above. The allocation sequence will be created by an investigator not involved in patient care ((J.L.), and the assignment of participants to interventions by a coordinator nurse at the radiological department who will be responsible for arranging the sessions. The primary investigators (A.P., P.T., K.K.) will be blinded to the intended treatment until the time of intervention; CRFA or CRYO will be applied to each patient after the opening of the sealed opaqued envelope inside the interventional radiological department. Clinical evaluation at 15 days (NPRS, PGIC), 4 weeks (NPRS, PGIC), 12 weeks (NPRS, PGIC, OKS, KOOS), and 24 weeks (NPRS, PGIC, OKS, KOOS) will be performed by an orthopedic resident (P.A.) not involved in the initial patient evaluation who will also be blinded to the applied intervention. Data analysists (J.L.) and outcome adjudicators will be also blinded during the implementation of the data. Unblinding will be performed in case of severe adverse effects (intense pain, hematoma, nerve damage etc.).

### Data collection and management

Data will be collected in paper forms at baseline and 2-, 4-, 12-, and 24-weeks post-intervention. Quality assessments and proper training sessions will be conducted to ensure that all information is collected properly. Outcome data include the NPRS, KOOS, OKS, and PGIC, all of which are patient-reported questionnaires. Demographic data, radiographs (downloaded from PACS and saved in JPEG format), and intraoperative pictures of skin marks or videos from the interventions will also be collected. A hard copy folder will be created for each patient, including all paper forms, demographic data, and intervention details. These folders will be kept safe in the hospital office of the primary investigator (A.P.) inside a special locker. The other main investigator (P.T.) will transfer all the data into an Excel database using the desktop computer at the office of the primary investigator (A.P.). Patient identification will be performed using only the national insurance number (11-digit number). Excel forms without patient names will be used for data analysis. Paper forms and computerized data could be assessed only by the two primary investigators. These files will be kept for at least three years after the last enrolled patient for future expansion of the research. We are not expecting participants to discontinue or deviate from the primary intervention. The coordinator nurse will be responsible for contacting them for missing appointments of clinical evaluation, either by phone or email. In case of refusal to attend, we plan to send the questionnaires by mail or make home visits. No other efforts will be made to minimize the loss of follow-up. Missing data from more than one time point will be a reason for dropout from the study.

Data entry, coding, security, storage, assessment of quality and creation of Excel tables will be under the responsibility of the two main investigators. Excel database would be password-protected. Personal information about potential and enrolled participants before, during, and after the trial will be collected to ensure confidentiality (using only the national insurance number as an indicator).

### Statistical methods

The sample size and power in the study have been previously described. Another statistical approach for the study is to evaluate all follow-up (post-intervention) measurements separately from the baseline. This may occur in two possible ways: one with the use of an ANCOVA procedure and the other with the use of ANOVA on the difference in the scores (follow-up minus the baseline). As Borm et al. [36] suggested, the ANCOVA approach requires a significantly smaller number of participants, reduced by a multiplicative factor that depends on the correlation estimate between the prior and posterior scores, which is usually high. More specifically, a significance level of 5% along with a power of 80% is achieved with 24–45 patients in total, when the correlation estimates between the a priori and a posteriori score is approximately 80–90%, which is a realistic approach. To sum up with the statistical plan, longitudinal (that is, repeated measures) analysis will be conducted with repeated measures ANOVA. Furthermore, the slopes of the score trajectories (trendlines) will be compared among the two patient groups using 1-way ANOVA. Bivariate comparisons for baseline versus follow-up will be conducted using ANCOVA. 1-way ANOVA will assist in detecting variations in bivariate differences of scores (baseline versus each follow-up) among the two patient groups. Data will be reported as differences between group means (means ± standard deviations, 95% CIs) if normal distributed, otherwise as medians with interquartile ranges. Categorical data will be reported as numbers and proportions. A statistically significant difference of at least two in NPRS scores between groups, will be interpreted as a minimal clinically important difference (MCID). The statistical method of pairwise (available case analysis—ACA) deletion will be used for missing data at only one time point. If more than two time points are missed the list-wise (Complete-case analysis—CCA) deletion method will be used. Statistical analysis will be performed using R language and Rstudio IDE, two well-known open-source products.

### Interim and additional analyses

Interim analyses will be performed when at least half of the CRFA and CRYO patients have been recruited; reasons for discontinuance would be serious complications or significant differences in clinical outcomes (especially for CRYO, which is a new approach not thoroughly tested so far). Although the sample size is small, we plan to perform additional subgroup analyses for age, sex, arthritis stage, and deformity type (varus or valgus).

## Discussion

Knee osteoarthritis is a common disease, yet the currently proposed conservative treatments have unsatisfactory overall efficacy and have been linked to an elevated risk of complications [7, 37–39]. This has prompted the need for effective non–pharmacological therapies to control chronic arthritic pain [40–43]. In this setting, new techniques such as CRFA and CRYO have been developed, aiming at nerve blockage to improve pain and disability caused by KOA [13, 16–18, 24, 44–46].

Radnovich et al. [13], in their multicenter, randomized, double-blind, sham-controlled trial, demonstrated that cryoneurolysis of the infrapatellar branch of the saphenous nerve (IPBSN) resulted in statistically significant decreased knee pain and improved symptoms compared to sham treatment for up to 150 days, while it appeared to be a safe and well-tolerated intervention. Mihalko et al. [16], in a single-center RCT study, assumed that preoperative cryoneurolysis of the superficial genicular nerves (IPBSN) and anterior femoral cutaneous nerve (AFCN) in patients with osteoarthritis would decrease postoperative opioid use relative to standard of care (SOC) treatment in patients undergoing TKA; compared with the SOC group, the cryoneurolysis group had improved functional scores and numerical improvements in pain scores across all follow-up assessments, with significant improvements observed in current pain from baseline to the 72-h and 2-week follow-up assessments. Finally, Nygaard et al. [14] recently presented a study protocol of a two-arm, parallel-group RCT, where 94 patients will be randomly allocated to a cryoneurolysis intervention group + standardized education and exercise or a sham group + standardized education and exercise. The target nerves would be the IPBSN and AFCN, and the primary outcome, the change in NPRS at 2 weeks. In our study, the CRYO group will be treated in the same manner as the CRFA group, and four genicular nerves will be targeted, with the SLGN and SMGN at two different areas.

In contrast to cryoneurolysis treatment, where the literature is scarce, the method of radiofrequency ablation for the treatment of symptomatic knee pain in osteoarthritic knees has been well documented in the past. In a cross-sectional survey, McCormick et al. [28] demonstrated a success rate of 35% based on a robust combination of outcome measures, and 19% of the procedures resulted in complete relief of pain at a minimum of six months of follow-up using the CRFA technique. They also demonstrated that 80% or greater relief from diagnostic blocks and a duration of pain of less than five years are associated with high accuracy in predicting treatment success. In their prospective, multicenter, randomized, crossover trial, Davis et al. [18] investigated the analgesic effect of CRFA in patients with knee osteoarthritis 12 months post-intervention and its ability to provide pain relief in patients who experienced unsatisfactory effects of intra-articular steroid injection. They demonstrated that at 12 months, 65% of the original CRFA group had pain reduction ≥ 50%, and the mean overall drop was 4.3 points (p < 0.0001) on the numeric rating scale, while 75% reported ‘improved’ effects. Hunter et al. [45] performed an extended evaluation of the patients enrolled in the study of Davis et al. [18] at 18- and 24-months post-intervention, showing a perceived positive effect with a mean NPRS score of 3.1 ± 2.7, and 3.6 ± 2.8, respectively. In another multicenter, randomized clinical trial, Chen et al. [17] compared the effectiveness of CRFA and a single injection of hyaluronic acid for the treatment of chronic knee pain; at 12-months, 65.2% of participants in the CRFA cohort reported ≥ 50% pain relief from baseline with a mean NPRS of 2.8 ± 2.4 (baseline 6.9 ± 0.8). Participants in the CRFA cohort also showed a 46.2% improvement in the total WOMAC score at the 12-month time point. Carlone et al. [46], in a recent retrospective review of 176 patients who underwent genicular nerve ablation, block, or both, found that 31.8% of the participants failed to respond to the block procedure, mainly due to the associated psychological comorbidities, smoking history, and diabetes. They also demonstrated that of the participants who underwent genicular nerve ablation, 53.7% reported less than 50% pain relief and 46.3% reported pain relief greater than or equal to 50% at the first follow-up visit.

Two recent systematic reviews have demonstrated promising results for the treatment of severe chronic knee pain using radiofrequency ablation, with minimal complications. Gupta et al. [47] reported positive patient outcomes in 17 studies (five RCTs) but inconsistent procedural methodology, patient assessment measures and small study sizes. In contrast, Ajrawat et al. [44], in their systematic review of 33 studies (13 RCTs) with 1,512 participants (mean age, 64.3 years, 32.5% males), found that in all studies (33/33), OA-related knee pain was alleviated from baseline until three to 12 months with RF modalities; six comparative studies reported > 50% pain relief in 65.5% and 19.3%, RF and control patients respectively.

It is apparent that there is an increasing body of evidence showing the effectiveness of CRFA and cryoneurolysis as treatment modalities for the osteoarthritic knee. Both techniques will be tested in the same manner in our study to investigate their capacity to control pain and disability in a mid-termed period of 6 months, while we will have the opportunity to record complication rates and failures. These treatment options may prove to be a significant aid for patients with KOA who are not at the end stage and for patients on waiting lists for surgery. Safety could also prove to be favorable as pharmacological treatments are associated with complications.

### Strengths and limitations

This will be a single-blinded randomized control trial with a follow-up period of six months. The strengths are the recruitment of enough patients to demonstrate a statistical significance of 90%, the precise targeting of the nerves in six different areas in accordance with old proven clinical reports and the new landmarks provided by recent anatomical studies and also that both techniques (CRFA and CRYO) would be applied at the same manner. Technical limitations also exist, as the effectiveness of these treatments relies heavily on accurate spotting of the genicular nerves and also from the fact that we are not able to spot all the nerves of the knee joint capsule. The extent of nerve damage and its capacity to regenerate also depends on parameters that cannot be fully controlled in this clinical trial. CRFA has a different mechanism of action (thermoablation with permanent? nerve damage) compared to CRYO that provokes cryoablation with Wallerian degeneration. This might reduce potential risks associated with the CRYO procedure but might also attenuate treatment effects. If CRYO turns out to be more effective or safer than CRFA would be probably considered as a novel non-pharmacological option for KOA that would be covered by medical insurances in the future.

## Conclusions

Cooled radiofrequency ablation and cryoneurolysis are two techniques that aim to block pain transmission in different ways; CRFA induces thermal nerve degradation, and cryoneurolysis causes Wallerian degeneration and subsequent analgesia, while the nerve retains its ability to regenerate. Spherical areas of ablation, time of procedure, targeting of genicular nerves and outcome evaluation are similar in both techniques. This will be the first instance where cryoneurolysis will be performed on the genicular nerves, in the same manner as the more investigated CRFA. The main goal is to demonstrate whether these techniques offer a stronger analgesic effect, while checking for and reporting potential side effects to better understand their role in the treatment of knee osteoarthritis.

## Data Availability

The primary investigators are responsible for data storage and availability upon request.

## Abbreviations

KOA: Knee OsteoArthritis
CRFA: Cooled RadioFrequency Ablation
RFA: Radiofrequency Ablation
CRYO: Cryoneurolysis
NPRS: Numerical Pain Rating Scale
KOOS: Knee Injury and Osteoarthritis Outcome Score
OKS: Oxford Knee Score
PGIC: 7-Point scale of Patient Global Impression of Change
SMGN: Superior Medial Genicular Nerve
SLGN: Superior Lateral Genicular Nerve
IMGN: Inferior Medial Genicular Nerve
IPBSN: Infrapatellar Branch of the Saphenous Nerve
MRGN: Medial Retinacular Genicular Nerve
AFCN: Anterior Femoral Cutaneous Nerve
RCT: Randomized Controlled Trial
SOC: Standard of Care treatment
NICE: National Clinical Guideline Centre
MCID: Minimal clinically important differences
AT: Adductor tubercle
